# Vedolizumab and new‐onset spondyloarthritis: Debunking the myth

**DOI:** 10.1002/ueg2.12624

**Published:** 2024-07-11

**Authors:** Joana Roseira, Irene Marafini, Nurulamin M. Noor

**Affiliations:** ^1^ Gastroenterology Department Unidade Local de Saúde do Algarve Portimão Portugal; ^2^ ABC ‐ Algarve Biomedical Center Faro Portugal; ^3^ Policlinico Universitario Tor Vergata Gastroenterology Unit Rome Italy; ^4^ Department of Gastroenterology Cambridge University Hospitals NHS Foundation Trust Cambridge UK; ^5^ Department of Medicine University of Cambridge School of Clinical Medicine Cambridge UK

**Keywords:** adverse effects, extra‐intestinal manifestations, inflammatory bowel disease, treatment

Despite an increased understanding of the etiopathogenesis of Inflammatory Bowel Disease (IBD), prevention or cure remains a distant aspiration and current treatment approaches often do not achieve long‐term disease remission.^1^ An additional complexity is that IBD can also be associated with a range of extra‐intestinal manifestations (EIMs).^2^ Among these EIMs, peripheral and axial rheumatological manifestations are perhaps the most prevalent, grouped under the term spondyloarthritis (SpA).^3^ The presentation of SpA can be before or after IBD diagnosis and presents several unique challenges. Notably, the presence of SpA can significantly impact on quality of life for patients and influence therapeutic decision‐making.^2^

Vedolizumab, a humanized IgG1 monoclonal antibody, targets the α4β7 integrin characteristically expressed by gut‐homing lymphocytes and is recognized by mucosal vascular addressin cell adhesion molecule 1 (MAdCAM1) on endothelial cells. The GEMINI‐1 and GEMINI‐2 trials, respectively demonstrated efficacy in inducing and maintaining remission in patients with UC and CD.^4^, ^5^ However, concerns have emerged regarding the potential for vedolizumab to induce or exacerbate EIMs, particularly SpA.^6^, ^7^ These concerns initially arose from case reports and small retrospective studies suggesting a possible temporal relationship between vedolizumab initiation and the onset or worsening of SpA symptoms. Given the significant impact of SpA on patient outcomes and quality of life, understanding the true risk associated with vedolizumab is crucial for making informed treatment decisions.

However, it is important to be aware of the limitations for such studies when attempting to draw conclusions about a possible causal relationship. First, retrospective studies typically have high risk of bias—notably selection bias, making them less ideal for establishing causality. Second, arthralgia and joint pain are distinct from joint inflammation, and previous reports did not use magnetic resonance imaging (MRI) to confirm the diagnosis of SpA after starting vedolizumab. Third, “new‐onset SpA” and “worsening of SpA symptoms” are clinically quite distinct scenarios, and no prior studies included imaging or rheumatology evaluations to rule out subclinical SpA before vedolizumab initiation. Fourth and crucially, previous reports suggesting vedolizumab induced new‐onset SpA typically included patients treated with TNF inhibitors (TNFi), which could have controlled subclinical arthropathy that then became apparent after withdrawal. Therefore, based on these above limitations, the attribution of a causal relationship between “new‐onset SpA” and vedolizumab may have been premature.

In this context, Rohekar et al. provide valuable insights by prospectively evaluating the incidence of new‐onset SpA in IBD patients initiating vedolizumab. This single‐center, observational study assessed 24 patients with active IBD (13 TNFi‐naive and 11 TNFi‐experienced patients), who had no prior history of arthritis or SpA. Patients were evaluated by a rheumatologist and underwent blinded MRI assessments at baseline, 8 weeks, and 24 weeks. The study found that neither the TNFi‐naïve, nor the TNFi‐experienced groups, developed new features of axial or peripheral SpA.

In this study, patients showed no evidence of new inflammatory back pain, enthesitis, dactylitis, or psoriasis over the 24‐week period. It is important to note that, the study employed rigorous methods, including a prospective design and serial MRI assessments, which were evaluated in a blinded manner by a central reader. The rheumatologist assessments were detailed, including several validated clinical outcome measures that were evaluated at each visit. Of note, the study's prospective nature allowed for temporal assessment of the effects from vedolizumab, and the use of blinded central readers for MRI assessments minimized interpretation bias. Finally, the comprehensive baseline assessments virtually excluded the possibility for subclinical SpA before initiating vedolizumab.

The comparatively small sample size in this study does limit the statistical power to detect rare events. However, the authors acknowledged this point and highlighted that this study was designed as a proof‐of‐principle based on feasibility. Moreover, formal sample size estimation or comparison with a historical group from the literature, was not possible given there had been no prior, prospective serial imaging study seeking to answer this question.

Finally, it is important to contextualize this study alongside recent systematic reviews and meta‐analyses. Indeed, a recent meta‐analysis found no significant difference in the frequency of new joint manifestations between either vedolizumab or other newly licensed medications.^8^ While another systematic review concluded that vedolizumab might actually reduce the occurrence of new EIMs.^9^

While further large‐scale, prospective investigation is needed to confirm these findings, this study helps provide reassuring data. As more treatment options emerge, the number of potential associations with adverse effects from any new therapy is likely to grow. While it is imperative to investigate any potential adverse association, we would caution against avoiding potentially effective medications for IBD, in the absence of strong causal evidence. Indeed, Rohekar et al. highlight the importance of evaluating any such association with prospective study designs and objective assessments to accurately evaluate the effects of treatment.

## CONFLICT OF INTEREST STATEMENT

Joana Roseira received speaking fee from Abbvie and Janssen; Irene Marafini received speaking fees from Galapagos, Abbvie; Nurulamin M. Noor received personal fees from BMS, Galapagos, Janssen, Lilly, SBK Healthcare, Takeda; and grants from Celltrion, Dr Falk, Pfizer, Pharmacosmos, Tillotts Pharma.

## Data Availability

The data presented in this study are available on request from the corresponding author.

## References

[1] Agrawal M , Jess T . Implications of the changing epidemiology of inflammatory bowel disease in a changing world. United Eur Gastroenterol J. 2022;10:1113–1120. 10.1002/ueg2.12317 PMC9752308 36251359

[2] Gordon H , Burisch J , Ellul P , Karmiris K , Katsanos K , Allocca M , et al. ECCO guidelines on extraintestinal manifestations in inflammatory bowel disease. J Crohn’s Colitis. 2024;18:1–37. 10.1093/ecco-jcc/jjad108 37351850

[3] Karreman MC , Luime JJ , Hazes JMW , Weel AEAM . The prevalence and incidence of axial and peripheral spondyloarthritis in inflammatory bowel disease: a systematic review and meta‐analysis. J Crohns Colitis. 2017;11:631–642. 28453761 10.1093/ecco-jcc/jjw199

[4] Feagan BG , Rutgeerts P , Sands BE , Hanauer S , Colombel JF , Sandborn WJ , et al. Vedolizumab as induction and maintenance therapy for ulcerative colitis. N Engl J Med. 2013;369(8):699–710. 10.1056/nejmoa1215734 23964932

[5] Sandborn WJ , Feagan BG , Rutgeerts P , Hanauer S , Colombel JF , Sands BE , et al. Vedolizumab as induction and maintenance therapy for Crohn’s disease. N Engl J Med. 2013;369(8):711–721. 10.1056/nejmoa1215739 23964933

[6] Varkas G , Thevissen K , De Brabanter G , Van Praet L , Czul‐gurdian F , Cypers H , et al. An induction or flare of arthritis and/or sacroiliitis by vedolizumab in inflammatory bowel disease: a case series. Ann Rheum Dis. 2017;76(5):878–881. 10.1136/annrheumdis-2016-210233 27899374

[7] Dubash S , Marianayagam T , Tinazzi I , Al‐Araimi T , Pagnoux C , Weizman AV , et al. Emergence of severe spondyloarthropathy‐related entheseal pathology following successful vedolizumab therapy for inflammatory bowel disease. Rheumatol. 2019;58(6):963–968. 10.1093/rheumatology/key267 30204909

[8] Tímár ÁE , Párniczky A , Budai A , Hernádfői MV , Kasznár E , Varga P , et al. Beyond the gut: a systematic review and meta‐analysis of advanced therapies for inflammatory bowel disease‐associated extraintestinal manifestations. J Crohn's Colitis. 2024;18(6):851–863. 10.1093/ecco-jcc/jjae002 38189533 PMC11147804

[9] Chateau T , Bonovas S , Le Berre C , Mathieu N , Danese S , Peyrin‐Biroulet L . Vedolizumab treatment in extra‐intestinal manifestations in inflammatory bowel disease: a systematic review. J Crohns Colitis. 2019;13(12):1569–1577. 10.1093/ecco-jcc/jjz095 31076751

